# Racial and Ethnic Differences in Barriers Faced by Medical College Admission Test Examinees and Their Association With Medical School Application and Matriculation

**DOI:** 10.1001/jamahealthforum.2023.0498

**Published:** 2023-04-14

**Authors:** Jessica Faiz, Utibe R. Essien, Donna L. Washington, Dan P. Ly

**Affiliations:** 1 National Clinician Scholars Program, Veterans Affairs (VA) Greater Los Angeles Healthcare System and UCLA, Los Angeles, California; 2 VA Health Services Research & Development Center for the Study of Healthcare Innovation, Implementation, and Policy, VA Greater Los Angeles Healthcare System, Los Angeles, California; 3 Division of General Internal Medicine and Health Services Research, Department of Medicine, David Geffen School of Medicine at UCLA, Los Angeles, California

## Abstract

**Importance:**

There has been disappointing progress in enrollment of medical students from racial and ethnic groups underrepresented in medicine, including American Indian or Alaska Native, Black, and Hispanic students. Barriers that may influence students interested in medicine are understudied.

**Objective:**

To examine racial and ethnic differences in barriers faced by students taking the Medical College Admission Test (MCAT).

**Design, Setting, and Participants:**

This cross-sectional study used survey data (surveys administered between January 1, 2015, to December 31, 2018) from MCAT examinees linked with application and matriculation data from the Association of American Medical Colleges. Data analyses were performed from November 1, 2021, to January 31, 2023.

**Main Variables and Outcomes:**

Main outcomes were medical school application and matriculation. Key independent variables reflected parental educational level, financial and educational barriers, extracurricular opportunities, and interpersonal discrimination.

**Results:**

The sample included 81 755 MCAT examinees (0.3% American Indian or Alaska Native, 21.3% Asian, 10.1% Black, 8.0% Hispanic, and 60.4% White; 56.9% female). There were racial and ethnic differences in reported barriers. For example, after adjustment for demographic characteristics and examination year, 39.0% (95% CI, 32.3%-45.8%) of American Indian or Alaska Native examinees, 35.1% (95% CI, 34.0%-36.2%) of Black examinees, and 46.6% (95% CI, 45.4%-47.9%) of Hispanic examinees reported having no parent with a college degree compared with 20.4% (95% CI, 20.0%-20.8%) of White examinees. After adjustment for demographic characteristics and examination year, Black examinees (77.8%; 95% CI, 76.9%-78.7%) and Hispanic examinees (71.3%; 95% CI, 70.2%-72.4%) were less likely than White examinees (80.2%; 95% CI, 79.8%-80.5%) to apply to medical school. Black examinees (40.6%; 95% CI, 39.5%-41.7%) and Hispanic examinees (40.2%; 95% CI, 39.0%-41.4%) were also less likely than White examinees (45.0%; 95% CI, 44.6%-45.5%) to matriculate at medical school. Examined barriers were associated with a lower likelihood of medical school application and matriculation (eg, examinees having no parent with a college degree had lower odds of applying [odds ratio, 0.65; 95% CI, 0.61-0.69] and matriculating [odds ratio, 0.63; 95% CI, 0.59-0.66]). Black-White and Hispanic-White disparities in application and matriculation were largely accounted for by differences in these barriers.

**Conclusions and Relevance:**

In this cross-sectional study of MCAT examinees, American Indian or Alaska Native, Black, and Hispanic students reported lower parental educational levels, greater educational and financial barriers, and greater discouragement from prehealth advisers than White students. These barriers may deter groups underrepresented in medicine from applying to and matriculating at medical school.

## Key Points

**Question:**

Are there racial and ethnic differences in barriers faced by Medical College Admission Test examinees, and are these differences associated with medical school application and matriculation rates?

**Findings:**

In this cross-sectional study of 81 755 examinees, American Indian or Alaska Native, Black, and Hispanic examinees had lower parental educational levels and greater financial and educational barriers (eg, outstanding premedical loans) than White examinees. These barriers were associated with lower likelihood of applying to and matriculating at medical school and largely accounted for racial and ethnic differences in application and matriculation.

**Meaning:**

Findings suggest that these barriers may deter groups underrepresented in medicine from applying to and matriculating at medical school.

## Teaser

This cross-sectional study of Medical College Admission Test examinees assesses potential racial and ethnic barriers for those interested in a medical career and the association of these barriers with medical school application and matriculation.

## Introduction

Increasing the racial and ethnic diversity of the physician workforce has been shown to improve quality of communication, patient satisfaction, and patient outcomes.^1,2,3,4,5,6^ However, although medical institutions have pledged to increase the diversity of the medical student body,^7^ little progress has been made toward this goal.^8,9,10^ In 2021, only 1% of medical school matriculants identified as American Indian or Alaska Native, 11% as Black, 13% as Hispanic, and 0.4% as Native Hawaiian or Pacific Islander^11,12^; these percentages remain low relative to their proportions in the US population.

Barriers upstream of matriculation may contribute to low representation. Explicit and implicit policies, such as residential redlining, school segregation, and mass incarceration, have disproportionately affected American Indian, Alaska Native, Black, and Hispanic communities.^13,14,15^ As Nguemeni Tiako et al^16^ and others^17,18,19,20,21,22,23^ have written, this structural racism leads to several barriers for groups underrepresented in medicine, including differences in parental educational level and resources and differences in the networks and informal knowledge required to, among other things, secure clinical shadowing and other extracurricular educational opportunities valued in the medical school application process. Interpersonal discrimination creates yet another barrier whose influence can occur anywhere along the process.^16,24^ These barriers may help explain why groups underrepresented in medicine are more likely to declare in high school their intentions of becoming a physician than they are to apply to or matriculate at medical school.^25^ Medical College Admission Test (MCAT) examinees, by virtue of intensely preparing for, paying for, and taking a rigorous examination, have demonstrated strong interest in a career in medicine. Studying MCAT examinees may provide a better understanding of the barriers students from racial and ethnic groups underrepresented in medicine face even before applying to and matriculating at medical school. We are not aware of prior literature that comprehensively studies these barriers among MCAT examinees.

In this study, we used data on MCAT examinees from the Association of American Medical Colleges (AAMC) to assess racial and ethnic differences in potential barriers for those interested in a medical career. We first examined parental educational level, which we use as a proxy for wealth. We examined financial and educational barriers that may reflect differences in resources. We then examined differences in extracurricular educational opportunities and in the influence of prehealth advisers (advisers for prospective medical students). Finally, we examined the association of these barriers with subsequent application to and matriculation at medical school and whether differences in these barriers helped explain racial and ethnic differences in medical school application and matriculation.

## Methods

### Data Sources and Study Population

We performed a cross-sectional study using deidentified data from the AAMC’s Post-MCAT Questionnaire (PMQ) and from the AAMC’s MCAT End of Day Survey (EOD).^26^ We used surveys administered between January 1, 2015, to December 31, 2018. The EOD is completed the same day after finishing the MCAT, and the PMQ is emailed by the AAMC to MCAT examinees within 4 days of completing the MCAT. Overall, 35% to 40% of MCAT examinees complete the PMQ each year^27^ (>99% complete the EOD). Data on respondents to the PMQ and to the EOD were linked by the AAMC to the AAMC Applicant Matriculant Data File as of October 1, 2020, which are data on who ultimately applied to and matriculated at a US doctor of medicine degree–granting medical school. This study was determined to be exempt from review by the University of California, Los Angeles Institutional Review Board because data were deidentified; therefore, informed consent was not required. This study followed the Strengthening the Reporting of Observational Studies in Epidemiology (STROBE) reporting guideline.

### Examinee Covariates

Our main variable of interest was race and ethnicity, which were self-identified by survey respondents. Data on race and ethnicity, which were complete for 99.4% of the potential sample, were limited by the AAMC to US citizens and permanent residents. To create mutually exclusive groups, we did not include in our analyses those who selected more than 1 race and ethnicity category (8997 respondents across 2015 to 2018). We did not include Native Hawaiian or other Pacific Islander respondents because of their small sample size (77 respondents across 2015 to 2018). The 5 groups examined were American Indian or Alaska Native; Asian; Black or African American; Hispanic, Latino, or of Spanish origin (Hispanic); and White. Individuals who self-identified as White in the survey were used as the reference group, given our interest in understanding barriers experienced by students from racial and ethnic groups underrepresented in medicine. Other examinee covariates included sex and age (categorized by the AAMC as <20, 20 or 21, 22 or 23, or >23 years).

### Key Independent Variables and Outcome Measures

There were 4 domains of variables hypothesized a priori as barriers (Table 1).^16,28^ The first is parental educational level, which we examined as a proxy for wealth.^20^ For this proxy, we used the AAMC variable of not having a parent with a bachelor’s degree.^29^ The second domain reflects financial and educational barriers to a career in medicine. The variables in this domain included attending a low-resourced college,^30^ having premedical education loans, reporting difficulty affording MCAT preparation materials, and taking a private MCAT preparation course. The third domain reflects extracurricular educational opportunities. Variables in this domain included participating in a middle school or high school premedical program, having a college laboratory experience, and shadowing a physician. The fourth reflects interpersonal discrimination. The variable in this domain was reporting that a prehealth adviser had a negative influence on their decision to pursue a career in medicine. Further details regarding these variables, including the survey and survey questions they are derived from, are given in Table 1. More details on the study methods are given in the eMethods in Supplement 1.

**Table 1.** Variables Reflecting Barriers Faced by MCAT Examinees by Domain

Variable	Survey	Survey question or variable definition
**Variable reflecting parental educational level**		
Parents with less than college degree	Post-MCAT Questionnaire	Less than a college degree (EO-1)^a^
**Variables reflecting financial and educational barriers**		
Low-resourced college	Derived from AAMC data on undergraduate institution	Fewer resources^b^
Outstanding premedical loans	Post-MCAT Questionnaire	Do you (or will you) have any outstanding education loans for your college/premedical education?
Difficulty affording preparation materials	Post-MCAT Questionnaire	What kinds of challenges, if any, have you had getting ready for the MCAT exam?: difficulty affording preparation courses and materials
Private MCAT course	MCAT End-of-Day Survey	How did you prepare for the examination you took today?: took an MCAT preparation course provided by a private company
**Variables reflecting extracurricular activities**		
Participated in middle or high school premedical program	Post-MCAT Questionnaire	Please indicate any experiences in which you have participated: middle school premedical or science program (eg, magnet science middle school, summer medical science program) and/or classroom-based summer, after-school, or Saturday premedical program for high school students
Participated in college laboratory program	Post-MCAT Questionnaire	Please indicate any experiences in which you have participated: laboratory research apprenticeship for college students
Shadowed a physician	Post-MCAT Questionnaire	Please indicate any experiences in which you have participated: shadowed a physician or other health care professional
**Variable reflecting interpersonal discrimination**		
Prehealth advisor negative about pursuing medicine	Post-MCAT Questionnaire	Please indicate the extent to which the following individuals have positively or negatively influenced your decision to pursue a career in medicine: prehealth advisor (selecting “very negative” or “somewhat negative”)

Abbreviations: AAMC, Association of American Medical Colleges; EO, education occupation; MCAT, Medical College Admission Test.

^a^ Determination of the socioeconomic status EO indicator is based on having both education and occupation information for at least 1 parent. If a respondent provided complete information for 2 parents, the EO indicator displayed is for the parent with the higher value.

^b^ Institutions that used the least selective admissions practices (they accepted students with a wide range of scores from college admissions tests) and had primarily nonresidential campuses (<25% of undergraduate students lived on campus and/or were enrolled full time) were classified as having fewer resources.

The study had 2 outcome measures. These outcome measures were whether the MCAT examinee applied to a US medical school and whether the examinee matriculated at a US medical school.

### Statistical Analysis

First, we examined unadjusted differences by race and ethnicity in barriers faced and in medical school application and matriculation. Second, to examine adjusted differences by race and ethnicity in these measures, we performed multivariable logistic regressions of each measure with race and ethnicity, controlling for age, sex, and year of examination. We used an available case analysis to address missing values of measures. We present adjusted results by race and ethnicity using marginal standardization (also known as predictive margins), holding other covariates at their mean values. Third, to examine whether these barriers were associated with application to and matriculation at medical school, we performed multivariable logistic regressions of application and of matriculation as a function of barriers. (We examined for collinearity among measures within each domain; these correlation matrices are given in eTable 1 in Supplement 1.) We estimated 2 models: the first model included race and ethnicity, age, sex, and year of examination, whereas the second model included these variables along with our measures of barriers. We presented these results as odds ratios (ORs). Analyses examining matriculation included all examinees and were not limited to those who applied. *P* values were calculated using *t* tests for means and χ^2^ tests for categories. A 2-sided *P* < .05 was considered statistically significant. All analyses were performed from November 1, 2021, to January 31, 2023, using Stata, version 17.0 (StataCorp LLC).

## Results

Our sample included 81 755 MCAT examinees between 2015 and 2018 (Table 2). Overall, 0.3% were American Indian or Alaska Native, 21.3% were Asian, 10.1% were Black, 8.0% were Hispanic, and 60.4% were White; 56.9% were female and 43.1% were male. Compared with 19.1% of Asian examinees and 25.3% of White examinees, 47.5% of American Indian or Alaska Native examinees, 42.2% of Black examinees, and 35.2% of Hispanic examinees were older than 23 years. Table 2 also presents unadjusted values of the key independent variables by race and ethnicity.

**Table 2.** Characteristics of MCAT Examinees by Race and Ethnicity, 2015-2018

Characteristic	Race and ethnicity, No. (%)						*P* value for difference between groups^a^			
	All (N = 81 755)	American Indian or Alaska Native (n = 221 [0.3%])	Asian (n = 17 381 [21.3%])	Black (n = 8221 [10.1%])	Hispanic (n = 6572 [8.0%])	White (n = 49 360 [60.4%])	American Indian or Alaska Native and White	Asian and White	Black and White	Hispanic and White
Sex										
Male	35 207 (43.1)	100 (45.2)	7331 (42.2)	2455 (29.9)	2713 (41.3)	22 608 (45.8)	.87	<.001	<.001	<.001
Female	46 548 (56.9)	121 (54.8)	10 050 (57.8)	5766 (70.1)	3859 (58.7)	26 752 (54.2)				
Age group, y										
<20	1158 (1.4)	1 (0.5)	600 (3.5)	80 (1.0)	37 (0.6)	440 (0.9)	<.001	<.001	<.001	<.001
20-21	34 673 (42.4)	54 (24.4)	8725 (50.2)	2178 (26.5)	1952 (29.7)	21 764 (44.1)				
22-23	24 219 (29.6)	61 (27.6)	4738 (27.3)	2490 (30.3)	2267 (34.5)	14 663 (29.7)				
>23	21 705 (26.6)	105 (47.5)	3318 (19.1)	3473 (42.2)	2316 (35.2)	12 493 (25.3)				
Variable reflecting parental educational level										
Parents with less than college degree	19 001 (25.2)	87 (42.6)	3599 (23.1)	2750 (37.9)	2957 (48.6)	9608 (20.7)	<.001	<.001	<.001	<.001
Variables reflecting financial and educational barriers										
Low-resourced college	2550 (3.3)	13 (6.6)	401 (2.4)	250 (3.2)	698 (11.1)	1188 (2.5)	<.001	.31	<.001	<.001
Outstanding premedical loans	29 170 (43.0)	96 (50.8)	4323 (32.1)	4363 (66.9)	2606 (48.5)	17 782 (42.0)	.01	<.001	<.001	<.001
Difficulty affording preparation materials	29 563 (42.5)	104 (53.9)	5020 (35.9)	3285 (48.5)	2893 (51.7)	18 261 (42.4)	.001	<.001	<.001	<.001
Private MCAT course	32 589 (43.0)	61 (29.5)	6918 (44.0)	2949 (38.8)	2467 (41.0)	20 194 (43.7)	<.001	.49	<.001	<.001
Variables reflecting extracurricular educational opportunities										
Participated in middle or high school premedical program	18 278 (23.3)	56 (25.7)	5461 (33.5)	2449 (31.0)	1485 (23.6)	8827 (18.5)	.006	<.001	<.001	<.001
Participated in college laboratory program	40 797 (52.0)	112 (51.4)	9539 (58.5)	3462 (43.9)	3064 (48.6)	24 620 (51.6)	.96	<.001	<.001	<.001
Shadowed a physician	67 800 (86.4)	186 (85.3)	13 834 (84.9)	6482 (82.1)	4943 (78.4)	42 355 (88.7)	.12	<.001	<.001	<.001
Variable reflecting interpersonal discrimination										
Prehealth adviser negative about pursuing medicine	10 502 (20.6)	47 (33.8)	2101 (21.3)	1181 (23.9)	846 (23.4)	6327 (19.5)	<.001	<.001	<.001	<.001
Application to and matriculation at a US doctor of medicine degree–granting medical school										
Applied	64 399 (78.77)	162 (73.3)	13 910 (80.0)	6223 (75.7)	4579 (69.7)	39 525 (80.1)	.01	.90	<.001	<.001
Matriculated	35 908 (43.9)	85 (38.5)	7782 (44.8)	3007 (36.6)	2475 (37.7)	22 559 (45.7)	.03	.03	<.001	<.001

Abbreviation: MCAT, Medical College Admission Test.

^a^ *P* values were calculated using *t* tests for means and χ^2^ tests for categories.

### Differences in Variables Reflecting Parental Educational Levels and Financial and Educational Barriers

Table 3 presents values of the key independent variables and outcomes by race and ethnicity, adjusted for demographic characteristics and examination year (corresponding adjusted ORs are in eTable 2 in Supplement 1). Overall, 39.0% (95% CI, 32.3%-45.8%) of American Indian or Alaska Native examinees, 23.9% (95% CI, 23.3%-24.6%) of Asian examinees, 35.1% (95% CI, 34.0%-36.2%) of Black examinees, and 46.6% (95% CI, 45.4%-47.9%) of Hispanic examinees reported having no parent with a college degree, compared with 20.4% (95% CI, 20.0%-20.8%) of White examinees (all stated differences statistically significant compared with White examinees). A total of 5.3% (95% CI, 2.5%-8.1%) of American Indian or Alaska Native examinees and 9.8% (95% CI, 9.0%-10.6%) of Hispanic examinees were from low-resourced colleges compared with 2.4% (95% CI, 2.3%-2.5%) of White examinees. Black examinees (63.8%; 95% CI, 62.6%-65.0%) and Hispanic examinees (45.7%; 95% CI, 44.4%-47.1%) were more likely than White examinees (42.0%; 95% CI, 41.5%-42.5%) to have outstanding premedical education loans, whereas Asian examinees were less likely (33.5%; 95% CI, 32.7%-34.3%).

**Table 3.** Adjusted Values for Key Independent Variables and Outcomes by Race and Ethnicity Among MCAT Examinees, 2015-2018^a^

Variable	Race and ethnicity, % (95% CI)				
	American Indian or Alaska Native	Asian	Black	Hispanic	White
Variable reflecting parental educational level					
Parents with less than college degree	39.0 (32.3-45.8)	23.9 (23.3-24.6)	35.1 (34.0-36.2)	46.6 (45.4-47.9)	20.4 (20.0-20.8)
Variables reflecting financial and educational barriers					
Low-resourced college	5.3 (2.5-8.1)	2.4 (2.2-2.6)	2.7 (2.4-3.0)	9.8 (9.0-10.6)	2.4 (2.3-2.5)
Outstanding premedical loans	45.9 (38.8-53.0)	33.5 (32.7-34.3)	63.8 (62.6-65.0)	45.7 (44.4-47.1)	42.0 (41.5-42.5)
Difficulty affording preparation materials	52.0 (45.0-59.1)	36.6 (35.8-37.4)	45.5 (44.3-46.7)	50.3 (49.0-51.6)	42.7 (42.2-43.1)
Private MCAT course	30.1 (23.8-36.4)	43.6 (42.8-44.4)	38.8 (37.7-39.9)	41.4 (40.1-42.6)	43.6 (43.1-44.1)
Variables reflecting extracurricular educational opportunities					
Participated in middle or high school premedical program	28.0 (21.7-34.3)	31.8 (31.1-32.5)	32.5 (31.4-33.6)	24.5 (23.4-25.6)	18.1 (17.7-18.4)
Participated in college laboratory program	55.8 (49.0-62.5)	57.1 (56.3-57.9)	47.0 (45.9-48.1)	50.7 (49.4-51.9)	51.2 (50.8-51.7)
Shadowed a physician	86.6 (82.3-90.9)	84.8 (84.2-85.3)	82.9 (82.1-83.7)	79.1 (78.1-80.1)	88.9 (88.6-89.2)
Variable reflecting interpersonal discrimination					
Prehealth advisor negative about pursuing medicine	29.9 (22.5-37.4)	21.6 (20.8-22.5)	20.7 (19.6-21.8)	21.1 (19.8-22.4)	19.3 (18.9-19.8)
Application to and matriculation at a US doctor of medicine degree–granting medical school					
Application	75.6 (70.0-81.1)	79.6 (79.0-80.2)	77.8 (76.9-78.7)	71.3 (70.2-72.4)	80.2 (79.8-80.5)
Matriculation	43.2 (36.2-50.1)	42.1 (41.4-42.9)	40.6 (39.5-41.7)	40.2 (39.0-41.4)	45.0 (44.6-45.5)

Abbreviation: MCAT, Medical College Admission Test.

^a^ Results were calculated using Association of American Medical Colleges data from 2015 to 2018. Adjusted probabilities were calculated using marginal standardization from logistic models of each outcome with race and ethnicity, also controlling for age, sex, and year of examination.

When examining how race and ethnicity were associated with MCAT preparation, 52.0% (95% CI, 45.0%-59.1%) of American Indian or Alaska Native examinees, 45.5% (95% CI, 44.3%-46.8%) of Black examinees, and 50.3% (95% CI, 49.0%-51.6%) of Hispanic examinees reported difficulty affording MCAT preparation materials compared with 42.7% (95% CI, 42.2%-43.1%) of White examinees. Compared with White examinees (43.6%; 95% CI, 43.1%-44.1%), American Indian or Alaska Native examinees (30.1%; 95% CI, 23.8%-36.4%), Black examinees (38.8%; 95% CI, 37.7%-39.9%), and Hispanic examinees (41.4%; 95% CI, 40.1%-42.6%) were less likely to take a private MCAT preparation course.

### Differences in Variables Reflecting Extracurricular Educational Opportunities

American Indian or Alaska Native examinees (28.0%; 95% CI, 21.7%-34.3%), Asian examinees (31.8%; 95% CI, 31.1%-32.5%), Black examinees (32.5%; 95% CI, 31.4%-33.6%), and Hispanic examinees (24.5%; 95% CI, 23.4%-25.6%) were more likely than White examinees (18.1%; 95% CI, 17.7%-18.4%) to participate in a middle school or high school premedical program (Table 3). Black examinees (47.0%; 95% CI, 45.9%-48.1%) were less likely to have a college laboratory experience than White examinees (51.2%; 95% CI, 50.8%-51.7%), whereas Asian examinees (57.1%; 95% CI, 56.3%-57.9%) were more likely. Asian examinees (84.8%; 95% CI, 84.2%-85.3%), Black examinees (82.9%; 95% CI, 82.1%-83.7%), and Hispanic examinees (79.1%; 95% CI, 78.1%-80.1%) were less likely than White examinees (88.9%; 95% CI, 88.6%-89.2%) to shadow a physician.

### Differences in Variable Reflecting Interpersonal Discrimination

Next, we examined our variable reflecting interpersonal discrimination. Overall, 29.9% (95% CI, 22.5%-37.4%) of American Indian or Alaska Native examinees, 21.6% (95% CI, 20.8%-22.5%) of Asian examinees, 20.7% (95% CI, 19.6%-21.8%) of Black examinees, and 21.1% (95% CI, 19.8%-22.4%) of Hispanic examinees reported that their prehealth adviser negatively influenced their decision to pursue medicine compared with 19.3% (95% CI, 18.9%-19.8%) of White examinees (Table 3).

### Differences in Application to and Matriculation at Medical School

Black examinees and Hispanic examinees were less likely to apply to medical school than White examinees. A total of 77.8% (95% CI, 76.9%-78.7%) of Black examinees and 71.3% (95% CI, 70.2%-72.4%) of Hispanic examinees applied to medical school compared with 80.2% (95% CI, 79.8%-80.5%) of White examinees (Table 3). Although American Indian or Alaska Native examinees did not differ statistically from White examinees in adjusted percentages applying to medical school, the wide 95% CI included potentially relevant lower point estimates. Asian examinees, Black examinees, and Hispanic examinees were less likely to matriculate at medical school than White examinees. A total of 42.1% (95% CI, 41.4%-42.9%) of Asian examinees, 40.6% (95% CI, 39.5%-41.7%) of Black examinees, and 40.2% (95% CI, 39.0%-41.4%) of Hispanic examinees matriculated at medical school compared with 45.0% (95% CI, 44.6%-45.5%) of White examinees.

### Association of Barriers With Application to and Matriculation at Medical School

Table 4 presents the association of barriers with application to medical school and the association of barriers with matriculation at medical school. In analyses for application to medical school, model 1, which controls for age, sex, and year of examination, shows that Black examinees (OR, 0.87; 95% CI, 0.82-0.92) and Hispanic examinees (OR, 0.61; 95% CI, 0.58-0.65) had lower odds of applying to medical school compared with White examinees. In model 2, which added our measures of barriers, those having lower parental educational levels, attending a low-resourced college, having outstanding loans, participating in a middle or high school program, and having a prehealth adviser who had a negative influence on the examinee pursuing medicine had lower odds of applying to medical school, whereas those taking a private MCAT course, participating in a college laboratory program, and shadowing a physician had higher odds of applying to medical school. After inclusion of these factors, the OR for Black examinees for application was 1.14 (95% CI, 1.04-1.24), and the OR for Hispanic examinees was 0.84 (95% CI, 0.77-0.93).

**Table 4.** Association of Barriers Faced With Application to and Matriculation at Medical School Among MCAT Examinees, 2015-2018^a^

Variable	Odds ratio (95% CI)			
	Application		Matriculation	
	Model 1	Model 2	Model 1	Model 2
Race and ethnicity				
American Indian or Alaska Native	0.76 (0.57-1.03)	0.85 (0.55-1.31)	0.93 (0.70-1.23)	0.83 (0.56-1.25)
Asian	0.97 (0.93-1.01)	1.02 (0.95-1.09)	0.89 (0.86-0.92)	0.86 (0.81-0.91)
Black	0.87 (0.82-0.92)	1.14 (1.04-1.24)	0.83 (0.79-0.88)	1.10 (1.02-1.19)
Hispanic	0.61 (0.58-0.65)	0.84 (0.77-0.93)	0.82 (0.78-0.86)	1.03 (0.94-1.12)
White	1 [Reference]	1 [Reference]	1 [Reference]	1 [Reference]
Variable reflecting parental educational level				
Parents with less than college degree	NA	0.65 (0.61-0.69)	NA	0.63 (0.59-0.66)
Variables reflecting financial and educational barriers				
Low-resourced college	NA	0.62 (0.54-0.70)	NA	0.50 (0.43-0.57)
Outstanding premedical loans	NA	0.74 (0.70-0.78)	NA	0.69 (0.66-0.72)
Difficulty affording preparation materials	NA	1.02 (0.96-1.07)	NA	0.99 (0.95-1.04)
Private MCAT course	NA	1.27 (1.20-1.34)	NA	1.13 (1.08-1.17)
Variables reflecting extracurricular educational opportunities				
Participated in middle or high school premedical program	NA	0.91 (0.85-0.96)	NA	1.03 (0.98-1.08)
Participated in college laboratory program	NA	1.70 (1.61-1.79)	NA	1.80 (1.73-1.88)
Shadowed a physician	NA	2.04 (1.90-2.19)	NA	1.75 (1.63-1.88)
Variable reflecting interpersonal discrimination				
Prehealth adviser negative about pursuing medicine	NA	0.77 (0.73-0.82)	NA	0.69 (0.66-0.73)

Abbreviations: MCAT, Medical College Admission Test; NA, not applicable.

^a^ Results were calculated using Association of American Medical Colleges data from 2015 to 2018. Model 1 controlled for age, sex, and year. Model 2 controlled for age, sex, and year, and additionally controlled for the listed variables reflecting barriers.

In adjusted analyses for matriculation, Asian examinees (OR, 0.89; 95% CI, 0.86-0.92), Black examinees (OR, 0.83; 95% CI, 0.79-0.88), and Hispanic examinees (OR, 0.82; 95% CI, 0.78-0.86) had lower odds of matriculating at medical school compared with White examinees. In model 2, having lower parental educational level, attending a low-resourced college, having outstanding loans, and having a prehealth adviser who had a negative influence on the examinee pursuing medicine were associated with lower odds of matriculating at medical school, whereas taking a private MCAT course, participating in a college laboratory program, and shadowing a physician were associated with higher odds of applying to medical school. After inclusion of these factors, the OR for Asian examinees was 0.86 (95% CI, 0.81-0.91), and the OR for Black examinees was 1.10 (95% CI, 1.02-1.19). The OR for Hispanic examinees was no longer statistically significantly different from 1.

## Discussion

We found meaningful disparities by race and ethnicity among MCAT examinees in barriers faced and in application to and matriculation at US medical schools. Black and Hispanic examinees were less likely to apply to medical school than White examinees, and Asian, Black, and Hispanic examinees were less likely to matriculate at medical school. Several factors related to parental educational level, financial and educational resources, extracurricular educational opportunities, and interpersonal discrimination—some more mutable than others —were independently associated with decreased odds of medical school application and matriculation. When examined collectively, we found that these barriers accounted for most or all of the disparities in medical school application and matriculation that we observed for Black and Hispanic MCAT examinees.^31^ For Hispanic examinees, accounting for these barriers reduced the disparity in applying to medical school from 39% lower odds to 16% lower odds, and it completely mitigated the disparity in matriculation. For Black examinees, adjusting for these barriers closed observed gaps in both application and matriculation.

We found that American Indian, Alaska Native, Asian, Black, and Hispanic examinees were more likely to have no parent with a bachelor’s degree and that Black and Hispanic examinees were more likely to have outstanding premedical loans. These findings are consistent with the hypothesis that socioeconomic disadvantage may underlie some of the differences in representation in the medical student body, which may result in financial barriers to preparing for the MCAT and applying to medical school.^32^ Differences in wealth may also explain why American Indian, Alaska Native, Black, and Hispanic examinees were more likely to report difficulty affording MCAT preparation materials and less likely to take a private MCAT course. Although closing this wealth gap is an enormously challenging issue, health systems can take a more active role in reducing this wealth gap.^15^ Additional interventions include broadening the benefits and criteria for the AAMC Fee Assistance Program, eliminating costly secondary applications, and continuing virtual interview options.^33^

Our findings regarding extracurricular educational opportunities were mixed. American Indian, Alaska Native, Asian, Black, and Hispanic examinees were more likely to have participated in a middle school or high school premedical program. This finding may suggest that programs such as the Health Careers Opportunity Program have been successful in identifying and exposing these students to science and medicine.^34^ Later, however, Asian, Black, and Hispanic examinees were less likely to shadow a physician, which may be due to lack of networks that would allow them to do so. Black examinees were also less likely to have a college laboratory experience. Lack of these clinical experiences may be detrimental when applying to medical school because they often signal a better understanding of the field.^35^ These findings inform the importance of further supporting premedical educational opportunities, such as postbaccalaureate programs and shadowing experiences, for underrepresented groups.^36,37^

We found that American Indian, Alaska Native, and Hispanic examinees were more likely to go to low-resourced colleges, and attending a low-resourced college was associated with lower odds of both applying to and matriculating at medical school. This finding may be due to several factors, including less rigorous application support and the emphasis placed on university prestige in the application process. Our results argue for increased efforts to focus recruitment at lower-resourced colleges.^38^ In addition, American Indian, Alaska Native, Asian, Black, and Hispanic examinees were significantly more likely to report that their prehealth adviser negatively influenced their decision to pursue a career in medicine. These findings raise serious concerns about the role that interpersonal discrimination plays among those in an important advisory position.^24,39^ Providing antiracism training to those involved in the medical school application process—from those in these advisory positions to members of admissions committees—may teach those in these important positions the enduring effects of structural racism and interpersonal discrimination that lead to these barriers in the pursuit of a career in medicine.^40,41,42,43^ In addition, admissions committees should use rubrics that specifically incorporate hardships applicants face, highlight the value that their diverse experiences bring, and use input from the communities that medical institutions serve in the selection process.^44,45,46^ Finally, our study demonstrates the continuing need for not only holistic but also race-conscious admissions processes.^47,48,49^

### Limitations

This study has limitations. Response rates to the PMQ were relatively low, and data were not available to compare respondents with nonrespondents. The PMQ respondents may represent a nonrandom sample of MCAT examinees, which may lend itself to sampling bias. On the basis of prior research of attrition from high school interest in medicine to medical school application and matriculation,^25^ our sample of MCAT examinees may not represent those with an interest in medicine but who did not take the MCAT, which likely underestimates our findings regarding differences by race and ethnicity in these barriers. The analysis was limited in its evaluation of structural racism, given that the survey does not directly ask questions about perceived discrimination and racism and used self-reported race and ethnicity as a proxy for the influence of racism. We did not include students who identified as Native Hawaiian or other Pacific Islander and those who selected more than 1 race and ethnicity, meaning we were unable to capture the perspectives of these students. The data did not include other aspects of examinees’ intersectional identities, such as sexual orientation, gender identity, and rurality.^50^ Our data did not capture other important variables, such as being a recipient of the fee assistance program and MCAT score. There are additional barriers to matriculation distinct from barriers to application that we were unable to capture, such as bias and discrimination among medical school interviewers and selection committees. Finally, as an observational study, residual confounding is possible.

## Conclusions

In this cross-sectional study, American Indian, Alaska Native, Black, and Hispanic examinees were more likely to face financial barriers and discouragement in their pursuit of a career in medicine than White examinees. These findings speak to how structural racism and interpersonal discrimination act to perpetuate the underrepresentation of students from these racial and ethnic groups in the medical school body and ultimately the physician workforce.

## Notes

Supplement 1. **eMethods**. Supplemental Details on Methods **eTable 1.** Correlation Matrices of Barriers Within Each Domain **eTable 2.** Adjusted Odds Ratios for Differences in Key Independent Variables by Race and Ethnicity Among MCAT Examinees, 2015-2018

Supplement 2.
Data Sharing Statement
