# Supplementary material for: Racial and Ethnic Differences in Barriers Faced by Medical College Admission Test Examinees and Their Association With Medical School Application and Matriculation
Source: JAMA Health Forum. 2023 Apr 14;4(4):e230498. doi: 10.1001/jamahealthforum.2023.0498 (PMC10105307; doi:10.1001/jamahealthforum.2023.0498)
Supplement: Supplement 1. — eMethods. Supplemental Details on Methods eTable 1. Correlation Matrices of Barriers Within Each Domain eTable 2. Adjusted Odds Ratios for Differences in Key Independent Variables by Race and Ethnicity Among MCAT Examinees, 2015-2018 [file jamahealthforum-e230498-s001.pdf]

Supplementary Online Content

Faiz J, Essien UR, Washington DL, Ly DP. Racial and ethnic differences in barriers faced by Medical College Admission Test examinees and their association with medical school application and matriculation. *JAMA Health Forum.* 2023;4(4):e230498. doi:10.1001/jamahealthforum.2023.0498

eMethods. Supplemental Details on Methods

eTable 1. Correlation Matrices of Barriers Within Each Domain

eTable 2. Adjusted Odds Ratios for Differences in Key Independent Variables by Race and Ethnicity Among MCAT Examinees, 2015-2018

This supplementary material has been provided by the authors to give readers additional information about their work.

© 2023 Faiz J et al. *JAMA Health Forum.*

eMethods. Supplemental Details on Methods

Examinees are given three weeks to complete the Post-MCAT Questionnaire (PMQ) to ensure that survey completion occurs before Medical College Admission Test (MCAT) score release. For respondents who took the MCAT more than once, the Association of American Medical Colleges (AAMC) provided data from the surveys after their most recent MCAT. We excluded the 228 examinees whose sex was missing or who declined to answer. Because the AAMC did not provide data on race and ethnicity for examinees who were not U.S. citizens or permanent residents, we excluded these 3,557 examinees. 2015 was chosen as the first year because a new version of the MCAT was created in 2015, and the AAMC could only provide End of Survey data (which contains the variable about taking a private MCAT course) beginning in 2015. The most current application and matriculation data at the time data was requested was through October 1, 2020, so the last year of data used was 2018 to allow for examinee matriculation by October 1, 2020.

© 2023 Faiz J et al. *JAMA Health Forum.*

eTable 1. Correlation Matrices of Barriers Within Each Domain

Variables reflecting financial and educational barriers

	Low-resourced college	Outstanding pre-medical loans	Difficulty affording preparation materials	Private MCAT course
Low-resourced college	1			
Outstanding pre-medical loans	0.008	1		
Difficulty affording preparation materials	0.029	0.233	1	
Private MCAT course	-0.022	-0.104	-0.111	1

Variables reflecting extracurricular educational opportunities

	Participated in middle or high school pre-medical program	Participated in college laboratory program	Shadowed a physician
Participated in middle or high school pre-medical program	1		
Participated in college laboratory program	0.112	1	
Shadowed a physician	0.032	0.080	1

Abbreviation: MCAT, Medical College Admission Test

© 2023 Faiz J et al. *JAMA Health Forum.*

eTable 2. Adjusted Odds Ratios for Differences in Key Independent Variables by Race and Ethnicity Among MCAT Examinees, 2015-2018

Variable	Race and ethnicity, % (95% CI)				
	**American Indian or Alaska Native**	**Asian**	**Black**	**Hispanic**	**White**
Variable Reflecting Parental Education					
Parents with less than college degree	2.49 (1.88-3.32)	1.23 (1.17-1.28)	2.11 (2.00-2.23)	3.41 (3.22-3.60)	1 (reference)
Variables Reflecting Financial and Educational Barriers					
Low-resourced college	2.27 (1.28-4.00)	0.99 (0.89-1.12)	1.12 (0.98-1.29)	4.40 (3.98-4.87)	1 (reference)
Outstanding pre-medical loans	1.17 (0.88-1.56)	0.70 (0.67-0.73)	2.44 (2.30-2.57)	1.16 (1.10-1.23)	1 (reference)
Difficulty affording preparation materials	1.46 (1.10-1.93)	0.78 (0.74-0.81)	1.12 (1.07-1.18)	1.36 (1.29-1.44)	1 (reference)
Private MCAT course	0.56 (0.41-0.75)	1.00 (0.96-1.04)	0.82 (0.78-0.86)	0.91 (0.86-0.96)	1 (reference)
Variables Reflecting Extracurricular Educational Opportunities					
Participated in middle or high school pre-medical program	1.76 (1.29-2.40)	2.11 (2.03-2.20)	2.18 (2.06-2.30)	1.47 (1.38-1.57)	1 (reference)
Participated in college laboratory program	1.20 (0.91-1.58)	1.27 (1.22-1.31)	0.84 (0.80-0.89)	0.98 (0.93-1.03)	1 (reference)
Shadowed a physician	0.81 (0.56-1.18)	0.70 (0.66-0.73)	0.61 (0.57-0.65)	0.47 (0.44-0.51)	1 (reference)
Variable Reflecting Interpersonal Discrimination					
Pre-health advisor negative about pursuing medicine	1.78 (1.25-2.55)	1.15 (1.09-1.22)	1.09 (1.01-1.17)	1.12 (1.03-1.21)	1 (reference)

© 2023 Faiz J et al. *JAMA Health Forum.*

Application and Matriculation					
Application	0.76 (0.57-1.03)	0.97 (0.93-1.01)	0.87 (0.82-0.92)	0.61 (0.58-0.65)	1 (reference)
Matriculation	0.93 (0.70-1.23)	0.89 (0.86-0.92)	0.83 (0.79-0.88)	0.82 (0.78-0.86)	1 (reference)

Abbreviation: MCAT, Medical College Admission Test

Note: Results were calculated using Association of American Medical Colleges data from 2015 to 2018. Logistic models of each outcome were performed with race and ethnicity, also controlling for age, sex, and year of examination. 95% confidence intervals in parentheses.

© 2023 Faiz J et al. *JAMA Health Forum.*
