# Supplementary material for: Racial and Ethnic Differences in Barriers Faced by Medical College Admission Test Examinees and Their Association With Medical School Application and Matriculation
Source: JAMA Health Forum. 2023 Apr 14;4(4):e230498. doi: 10.1001/jamahealthforum.2023.0498 (PMC10105307; doi:10.1001/jamahealthforum.2023.0498)
Supplement: Supplement 2. — Data Sharing Statement [file jamahealthforum-e230498-s002.pdf]

Data Sharing Statement

Faiz. Racial and Ethnic Differences in Barriers Faced by Medical College Admission Test Examinees and Their Association With Medical School Application and Matriculation. *JAMA Health Forum*. Published April 14, 2023. doi:10.1001/jamahealthforum.2023.0498

Data
Data available: No

Additional Information
Explanation for why data not available: The data are protected under a DUA with the AAMC.
